# A Fast Simulation Method for Evaluating the Single-Event Effect in Aerospace Integrated Circuits

**DOI:** 10.3390/mi14101887

**Published:** 2023-09-30

**Authors:** Xiaorui Zhang, Yi Liu, Changqing Xu, Xinfang Liao, Dongdong Chen, Yintang Yang

**Affiliations:** 1 Laboratory of Digital IC and Space Application, School of Microelectronics, Xidian University, Xi’an 710071, China; 2 Guangzhou Institute of Technology, Xidian University, Guangzhou 510555, China

**Keywords:** aerospace integrated circuits, single-event effect, multi-point fault injection method, grouping method

## Abstract

With the continuous progress in integrated circuit technology, single-event effect (SEE) has become a key factor affecting the reliability of aerospace integrated circuits. Simulating fault injection using the computer simulation technique effectively reflects the SEE in aerospace integrated circuits. Due to various masking effects, only a small number of faults will result in errors; the traditional method of injecting one fault in one workload execution is inefficient. The method of injecting multiple faults in one workload execution will make it impossible to judge which fault results in errors because the propagation characteristic of SEE and faults may affect each other. This paper proposes an improved multi-point fault injection method to improve simulation efficiency and solve the problems of the general multi-point fault injection method. If one workload execution does not result in errors, multiple faults can be verified by one workload execution. If one workload execution results in errors, a specific grouping method can be used to determine which faults result in errors. The experimental results show that the proposed method achieves a good acceleration effect and significantly improves the simulation efficiency.

## 1. Introduction

Due to the existence of the earth radiation belt proton, galactic cosmic ray, solar cosmic ray, and so on, the radiation effect in the space environment seriously affects the reliability of aerospace integrated circuits [1]. With the continuous progress in integrated circuit technologies, single-event effect (SEE) has become a key factor affecting the reliability of aerospace integrated circuits [2,3]. SEE results from the generation of electron-hole pairs in the sensitive area of the device when high-energy particles bombard microelectronic devices. These charges are collected by the electrodes of the sensitive device, affecting the working state of the aerospace integrated circuits [4,5,6]. The impact of SEE on aerospace integrated circuits will lead to system functional errors and even system functional failure in severe cases. So, it is necessary to evaluate the SEE in aerospace integrated circuits. 

The evaluation methods of SEE mainly include onboard experiments, ground experiments [7], and computer simulation techniques [8]. The experiment data obtained from onboard experiments and ground experiments can reflect realistic changes in aerospace integrated circuits in the radiation environment, but there are disadvantages, such as long test cycles and high costs. In contrast, computer simulation technology has the characteristics of low cost and high efficiency, which can be used as a supplement to onboard experiments and ground experiments.

The evaluation method of SEE based on computer simulation technique mainly includes device-level simulation method, circuit-level simulation method, and system-level simulation method according to the hierarchy. 

Device-level simulation method uses relevant parameters to build device models and then adds the radiation model to simulate the impact of SEE [9,10,11]. This method can reflect the physical mechanism of SEE with high accuracy, but the simulation speed is slow, so it is only suitable for small-scale circuits. Circuit-level simulation object is the circuit netlist described by SPICE. It needs to obtain the transient current source model generated by the device-level simulation result, and then the transient current source model is added to the circuit node by PWL or Verilog-A [12,13,14], etc. Although this method improves the simulation speed, it still cannot meet the speed requirements of large-scale digital circuits. So, it is suitable for analog circuits and small-scale digital circuits. System-level simulation method modifies the relevant signal to simulate the impact of SEE in the RTL model or higher hierarchy [15,16,17]. The simulation speed is fast, but it cannot reflect the physical mechanism of SEE and it cannot be compared to the SEE experiment result. 

**Figure 1:**
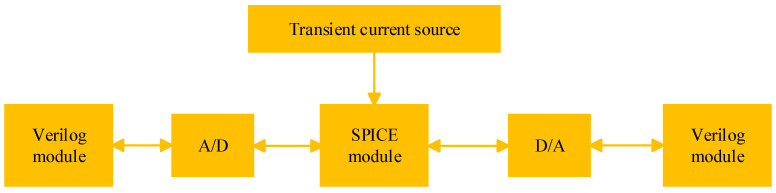
Mixture simulation method.

Therefore, this paper uses a mixture simulation method. The simulation object is divided into different modules according to the execution function. The module to add the transient current source model is described by SPICE; the other modules are described by RTL. Different types of modules are connected through A/D and D/A interfaces, as shown in Figure 1.

The transient current source model adopted in this paper is obtained from previous work [18] and is added by PWL.

With the development of integrated circuit technology, the fault injection set becomes larger and larger. To effectively reflect the SEE in aerospace integrated circuits, tens of thousands of faults need to be verified at least [19]. The traditional fault injection method injects only one fault in one workload execution. However, due to the propagation characteristics of SEE and the design characteristics of aerospace integrated circuits, most faults will not result in errors [20,21]. The traditional fault injection method is inefficient. 

Injecting multiple faults in one workload execution has the problem of being unable to judge which fault results in errors because the errors continue to propagate as the aerospace integrated circuits run. So, it will be impossible to judge whether the subsequent faults injected after errors occurred result in errors. In addition, injecting multiple faults in one workload execution may result in additional errors compared to injecting one fault.

The current research direction is mainly to improve efficiency by reducing the fault injection set, which is that some faults can be judged whether they result in errors without simulation. Some studies analyze SEE by analyzing the instruction program to determine whether faults will result in errors [22,23,24]. Some studies analyze from the perspective of logic masking [25,26], register reading, and writing [27].

Although these methods can directly improve simulation efficiency, all methods are based on injecting only one fault in one workload execution. Otherwise, the methods of analyzing the instruction program makes accuracy decline. The methods of analyzing logic masking consider logic masking without considering sequential circuits. The method of analyzing register reading and writing only considers the circuits with read and write ports. 

So, an improved multi-point fault injection method is proposed in this paper. This method not only solves the problems existing in the general multi-point fault injection method but also can be applied to any aerospace integrated circuits. The improved multi-point fault injection method injects multiple faults in one workload execution. If the workload execution does not result in errors, it is proved that the faults injected in this workload execution would not result in errors. If the workload execution results in errors, a grouping method is used to find faults that result in errors. Simulation results based on the Leon2 system show that the proposed method can achieve a speedup of 60.69× at most.

## 2. Proposed Method

### 2.1. Motivation

The general multi-point fault injection method cannot determine which fault is an error fault and the grouping method can solve the problem. The purpose of grouping is to find the error faults.

Once the workload execution results in errors, the injected faults will be grouped. If there are no errors, the injected faults would not be grouped. Each group corresponds to a workload execution with injected faults. Considering the propagation characteristics of SEE, determining whether a fault is an error fault can only be done on the premise that only one fault is injected in one workload execution. Therefore, the grouping ends until the workload execution does not result in errors or the number of injected faults in one workload execution is 1. 

Injecting multiple faults does result in additional errors compared to injecting one fault, even if the probability of this happening is low. However, the number of injected faults must be one when judging whether one fault is an error fault or not by using the grouping method, so this shows that even if it occurs, only increasing the number of workload executions will not affect the correctness of the results.

Based on the above analysis, the multi-point fault injection method based on the grouping method can solve the problems of general multi-point fault injection and improve the simulation efficiency.

### 2.2. The Introduction of One Multi-Point Fault Injection Process

**Figure 2:**
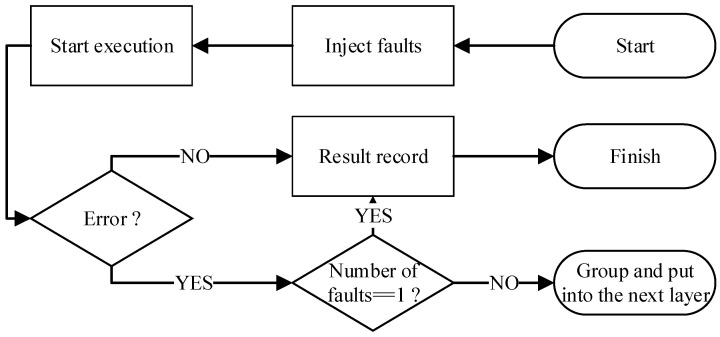
The process of deciding whether to group the injected faults.

Before analyzing SEE, it is necessary to run one workload execution completely without injecting faults to obtain the correct simulation data, called golden data, which is used to judge whether the workload execution that injected faults results in errors. The multi-point fault injection process proposed in this paper needs to inject a certain number of faults into one workload execution first; these faults are called the first layer faults, and this workload execution is called the first layer workload execution. The simulation results of the first layer workload execution will be compared with the golden data. The specific grouping method is obtained in Section 2.3. Each group is called one fault group. After the process of the first layer ends, the process of the second layer begins. First, it is necessary to judge the number of fault groups and the number of faults contained in each group in the second layer, which depends on the grouping method of the previous layer. A multi-point fault injection process ends until a layer contains no fault group. The process of deciding whether to group the injected faults is shown in Figure 2.

**Figure 3:**
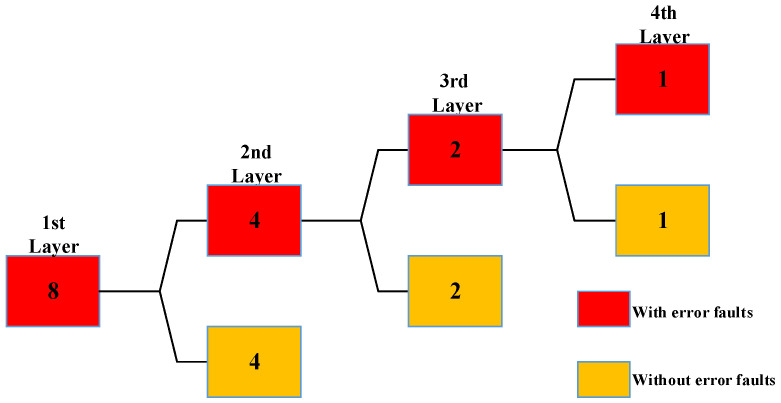
The structure of one multi-point fault injection process.

Take the example of injecting eight faults in one workload execution and the grouping method is to divide the faults as equally as possible into two groups. Assume that there is one and only one error fault in eight faults. The number of the first layer faults is eight. In the first layer, eight faults are injected in one workload execution. The comparison results are inconsistent, and the number of faults injected is greater than one, the eight faults will be equally divided into two groups of four faults each and are defined as the second layer fault groups. The second layer includes two fault groups, so the number of the second layer workload executions is two and one workload execution results in errors. The above process is repeated until the multi-point injection process ends. The structure of this multi-point fault injection process is shown in Figure 3. The number inside the rectangle indicates the number of faults injected into one workload execution. Red means the workload execution results in errors; yellow means the workload execution does not result in errors.

### 2.3. Determination of Relevant Parameters

**Figure 4:**
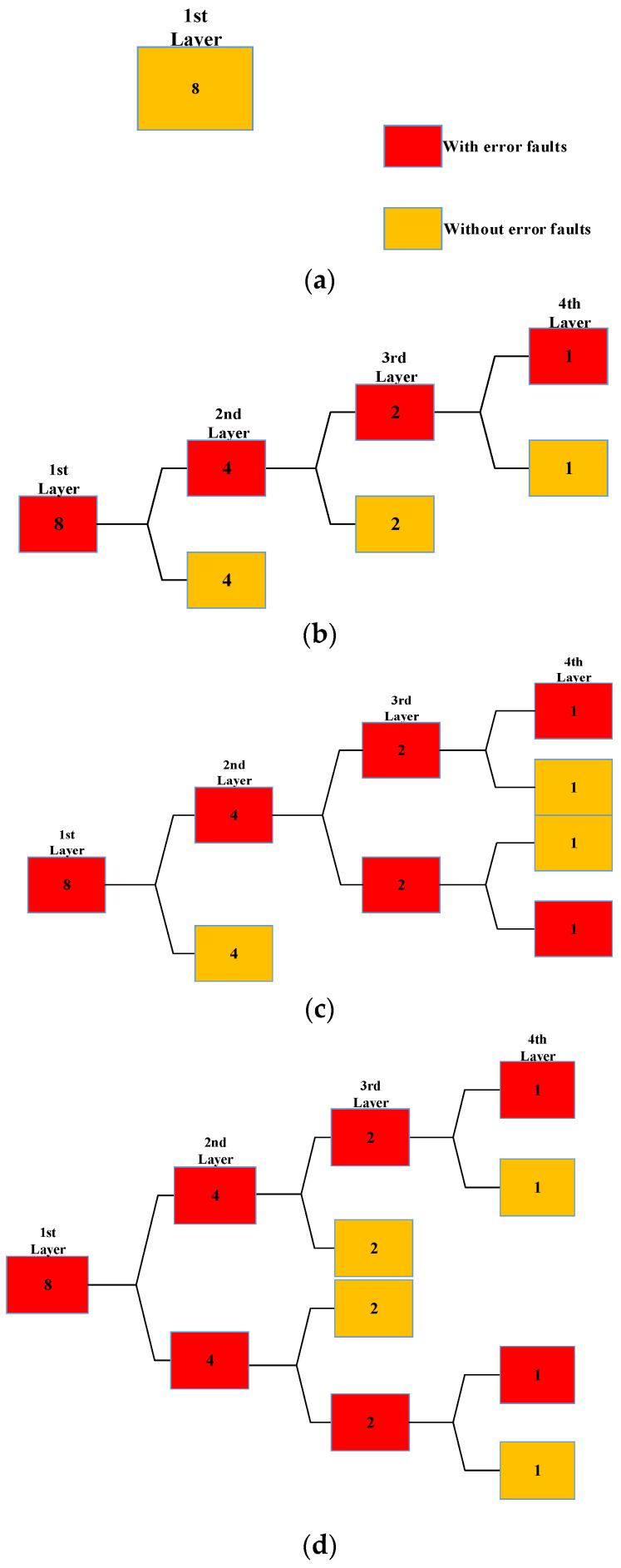
There are four structures and the number of the first layer faults is 8: (**a**) the number of error faults is 0 and the number of workload executions required is 1; (**b**) the number of error faults is 1 and the number of workload executions required is 7; (**c**) the number of error faults is 2 and the number of workload executions required is 9; (**d**) the number of error faults is 2 and the number of workload executions required is 11.

The four cases are shown in Figure 4. The number of the first layer faults in each case is eight. In Figure 4a, the first layer workload execution does not result in errors because there are no error faults. However, the number of workload executions required increases as the number of error faults increases. In Figure 4b–d, 7, 9 and 11, workload executions are required, and the corresponding numbers of error faults are 1, 2, and 2. When the number of error faults increases to a certain number, the proposed method will be less efficient than the traditional fault injection method.

From the above analysis, it can be known that it is necessary to control the number of error faults in the first layer faults, which is related to the error probability. Otherwise, the number of fault groups divide, and the number of faults contained in each fault group are different with different grouping methods. So, one multi-point fault injection process requires two input parameters: the number of the first layer faults and the specific grouping method. Next, this paper will explain how to choose the two parameters through theoretical analysis.

Assume that the number of the first layer faults is k, and the error probability is p. The number of error faults is a random variable with a binomial distribution. Therefore, the probability that the number of error faults = i is:

(1) $$P(x=i)=C_{k}^{i}\times p^{i}\times {\left(1-p\right)}^{k-i}$$

$C_{k}^{i}$ denotes the binomial coefficient. The number of error faults can vary from 0 to k theoretically, so the number of workload executions required for one multipoint fault injection process is:

(2) $$\mathrm{sum}=1+\sum_{i=1}^{k}\left[R(k,i)\times P\left(x=i\right)\right]$$

R (k, i) denotes the number of workload executions required when the number of the first layer faults is k, and the number of error faults is i. If i = 0, the number of workload executions required is one.

Then, it only needs to determine the grouping method to calculate R (k, i). To determine the grouping method, it is necessary to obtain the complete structure of this multi-point fault injection process. The complete structure refers to the number of layers, the number of fault groups contained in each layer, and the number of faults contained in each fault group. To obtain the complete structure, it is assumed that each workload execution will result in errors. Therefore, if the number of faults in one fault group is greater than 1, it will be grouped until the number of faults is 1 for all fault groups in one layer.

The grouping method in this paper only considers four grouping methods, which is dividing the faults in one fault group as equally as possible into two groups, three groups four groups, or five groups. Four complete structures can be obtained for the same number of the first layer faults.

Some special grouping situations need to be explained to ensure that the number of faults in each group is as equal as possible, for example, if the number of faults in one fault group is 7 and the faults are divided into three groups, the number of faults in the three group is 3, 2, and 2. In addition, if the number of faults is 3 but it needs to be divided into four groups, it will only be divided into three groups and each group contains one fault. 

The probability pro that one fault group contains error faults is:

(3) $$\mathrm{pro}=1-\frac{C_{k-n_{m_{h}}}^{i}}{C_{k}^{i}}$$

$C_{k}^{i}{\;\mathrm{and}\;C}_{n_{m_{h}}}^{i}\;$ denotes the binomial coefficient. $m_{h}$ denotes the m-th fault group of the h-th layer. $n_{m_{h}}$ denotes the number of faults contained in the m-th fault group of the h-th layer.

When a fault group contains error faults, it will be grouped and will need additional workload executions. The number of additional workload executions depends on the grouping method; if the fault group is divided into two groups, the number of additional workload executions is 2; if the fault group is divided into three groups, the number of additional workload executions is 3. The other grouping methods are the same. Summing up the number of additional workload executions required for all fault groups, the number of workload executions required can be obtained when the number of error faults is i (i ≥ 1), which is:

(4) $$R(k,i)=\sum_{h=1}^{H}\sum_{m=1}^{M_{h}}\left\{\left[1-\frac{C_{k-n_{m_{h}}}^{i}}{C_{k}^{i}}\right]\times group\right\}$$

H denotes the number of layers, $M_{h}$ denotes the number of fault groups at the h-th layer, and group denotes the number of additional workload executions required when a fault group contains error faults.

Therefore, the number of workload executions required for one multi-point fault injection process is shown:

(5) $$\begin{array}{ll} sum & =1+\sum_{i=1}^{k}\left[P\left(x=i\right)\times R\left(k,i\right)\right]\\ & =1+\sum_{i=1}^{k}\left\{P\left(x=i\right)\times \sum_{h=1}^{H}\sum_{m=1}^{M_{h}}\left\{\left[1-\frac{C_{k-n_{m_{h}}}^{i}}{C_{k}^{i}}\right]*group\right\}\right\} \end{array}$$

The speedup is shown:

(6) $$speedup=\frac{k}{sum}$$

The four grouping methods can obtain four speedups with the same number of the first layer faults. Based on the speedup, the optimal number of the first layer faults and the optimal grouping method can be selected. It is worth stating that the error probability also determines the upper limit for the number of the first layer faults. The error probability times the optimal number of the first layer faults should be less than one, because the case without error faults requires the least number of workload executions.

When error probability = 0, it indicates there are no error faults. To ensure the fastest speed, it is necessary to double the number of the first layer faults this time as the number of the first layer faults in the next multi-point fault injection process. When error probability = 0, the more the number of the first layer faults and the more faults can be verified in one workload execution.

### 2.4. Add Checkpoints to Optimize the Grouping Method

**Figure 5:**
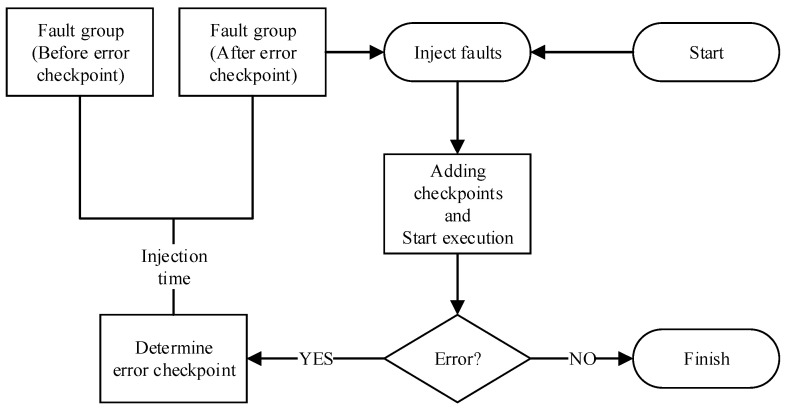
The process of determining error checkpoints.

After obtaining the optimal number of the first layer faults and the optimal grouping method in Section 2.3, this section proposes a method of adding checkpoints to optimize the grouping method. By judging whether the workload execution results in errors at all checkpoints, the earliest checkpoint that detects errors when the workload execution results in errors is called the error checkpoint. If one workload execution results in errors, it Is proved that the injection time of error faults is before the error checkpoint. All faults injected after the error checkpoint need to be injected into one workload execution to determine whether these faults contain error faults because of the propagation characteristics of SEE. For the faults injected after the error checkpoint, it requires the same process for determining other error checkpoints until the workload execution does not result in errors. The process of adding checkpoints is shown in Figure 5.

The faults are divided into multiple parts by error checkpoints, and the number of faults contained in each part can be obtained through the grouping method based on checkpoints. It is equal to obtaining the number of the first layer faults in each part, so that each part can search for error faults using the optimal grouping method mentioned in Section 2.3, and the number of error checkpoints depends on the number of error faults. 

**Figure 6:**
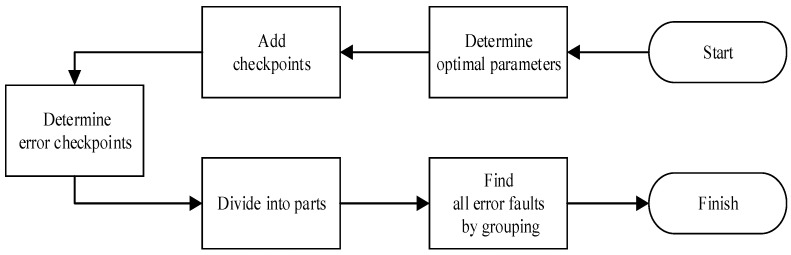
The process of one multi-point fault injection process with addition of checkpoints.

The process of one multi-point fault injection process with the addition of checkpoints is shown in Figure 6. Adding checkpoints is performed to find the injection time range of error faults, and the number of workload executions required can be reduced. Results show that the method with the addition of checkpoints is faster than the multi-point injection method without the addition of checkpoints.

### 2.5. Overall Simulation Flow

**Figure 7:**
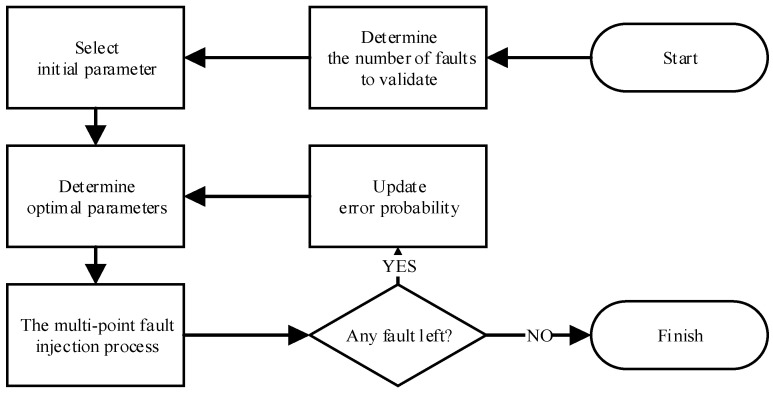
The overall simulation flow.

The overall simulation flow is shown in Figure 7. We can estimate the error probability and choose an appropriate initial value to satisfy the error probability times the optimal number of the first layer faults that should be less than one. So, the initial number of the first layer faults in the first multi-point fault injection process is chosen as 10 in this paper. The simulation flow ends until the number of faults that have been verified is large enough to calculate the error probability of the circuits. 

## 3. Results and Analysis

### 3.1. Simulation Setup

**Figure 8:**
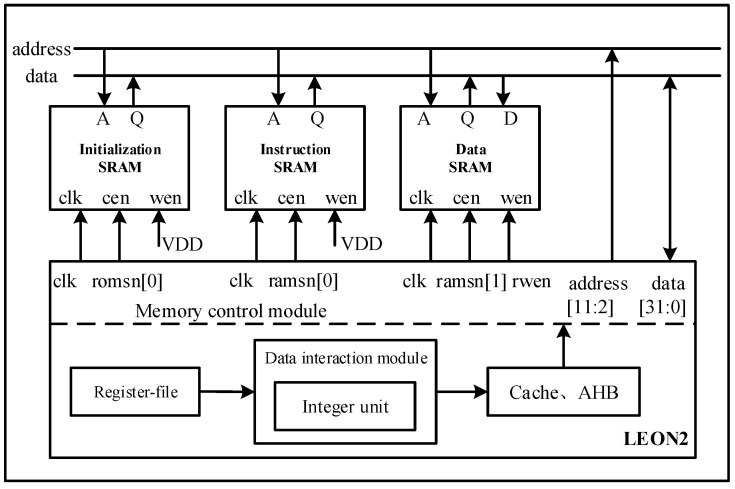
The structure of the Leon2 system.

To validate the proposed method, the SEE simulation based on the Leon2 system is carried out in this section. Leon2 processor is a 32-bit processor developed by the European Space Agency (ESA). Its code is composed of synthesizable VHDL code. The Leon2 system has one Leon2 processor and three SRAMs. The main functional modules of the Leon2 processor include an integer unit, register-file, cache, and memory controller module. Figure 8 shows the structure of the Leon2 system.

The current source model selected in this paper is based on the previous work and it is added to the drain of NMOSFET [28]. When faults are injected into the digital circuits, the following three conditions may occur in the digital circuits [29]: a. direct errors: the output data of the workload execution is wrong; b. potential errors: the output data of the workload execution is correct, but the operating state of the workload execution is wrong; c. invalid errors: errors occur but recover quickly.

This paper only considers direct errors and potential errors. When the workload execution is finished, if the information stored in the data SRAM is inconsistent, it is proved that direct errors occurred. If the five-stage pipeline registers state as inconsistent, it is proved that potential errors occurred. Either direct errors or potential errors are considered to indicate the workload execution results in errors.

The time interval between the two checkpoints chosen in this paper is the time for the aerospace integrated circuit system to complete one process from reading data to writing data, which is to ensure that the errors can be detected. The test program adopted in this paper is the matrix multiplication program. By analyzing the test program, the Leon2 system takes 28 clock cycles to complete one process from reading data to writing data. The Leon2 processor clock frequency is 100Mhz, so the time interval between two checkpoints is selected as 300 ns to round. Parameters related to one workload execution are shown in Table 1. 

From 0 to 8700 ns, it is the period of system initialization. No faults are injected in this period. The modules verified in this paper include integer unit, register-file, cache, and memory controller module. 

Table 2 shows the areas of the different modules obtained through the Design compile software. Considering that the register-file has a larger circuit area than other modules and makes the simulation results statistically significant [19], the other modules inject 10,000 faults each and the register-file module injects 100,000 faults. 

### 3.2. Result and Analysis

This paper ensures that the time and location of the fault injection are consistent when comparing the speed improvement with and without checkpoints. Table 3 shows the error probability of different modules and the speed improvements with and without checkpoints, which is compared with the traditional fault injection method. The results show that the proposed method can achieve a speedup of 60.69× at most and the method of adding checkpoints can further improve the simulation efficiency. 

While the traditional fault injection method is equivalent to verifying one fault by one workload execution, the proposed method equals reducing the required number of workload executions by the grouping method in Section 2.3. In the initial multi-point fault injection processes, the number of verified faults is small, and the error probability is unstable. After the error probability is stable, the number of the first layer faults and the grouping method will not change until the end of the simulation process. Table 4 shows the optimal number of the first layer faults, the optimal grouping method for different injection modules, and the speedup by theoretical analysis after the error probability is stable. 

As can be seen from Table 3 and Table 4, the optimal number of the first layer faults times error probability is less than 1 and the optimal grouping method of the four modules is tripartition. The speedup obtained by simulation results is smaller than that obtained by theoretical analysis. Firstly, the error probability is unstable in the initial multi-point fault injection processes, which means speedup is unstable. Secondly, it takes some time to calculate the optimal number of the first layer faults and the grouping method before starting a multi-point fault injection process. Injecting multiple faults also increases the time to execute one workload execution compared to injecting one fault. However, the calculation time and additional time added have little impact compared to the time of executing one workload execution. Lastly, the higher the number of faults injected in one workload, the higher the probability of resulting in additional errors. It indicates more workload executions are needed to exclude additional errors. The more faults injected in one workload execution, the more the speedup decreases compared with the theoretical analysis.

Table 3 also shows that the simulation with addition of checkpoints is more efficient than without checkpoints because error checkpoints reduce the search range by dividing the fault into several parts according to the injection time. Table 5 shows the number of faults that need to be searched under different numbers of error faults with and without addition of checkpoints after the error probability is stable. 

Adding multiple checkpoints does increase the time of executing one workload execution, but the method proposed in this paper only adds multiple checkpoints when determining error checkpoints. After grouping based on error checkpoints, there is no need to add checkpoints. In addition, determining one error checkpoint means that an additional workload execution is needed to determine whether the faults injected after the error checkpoint contain error faults, but the increase in the number of simulations is less than the decrease in the number of simulations compared to the decrease in the search range. The results in Table 3 show that the increased time in addition of checkpoints is less than the decreased time.

The speed improvement in the method with checkpoints is not significantly increased compared to the method without checkpoints because the number of the first layer faults is selected according to error probability. Therefore, the promotion after adding checkpoints depends on the probability that the error fault = 0 occurs. Table 6 shows the probabilities of different numbers of error faults.

Table 3 shows that speedup depends on error probability and the lower the error probability, the greater the speed improvement. The integer unit has the highest error probability than other modules because the five-stage pipeline registers state of the Leon2 processor is mainly determined by the integer unit. The register-file module has the lowest error probability; it is related to the number of registers used by the test program.

In this study, we evaluated the Leon2 system by running the matrix multiplication program. The method proposed in this paper can be applied to other processors and programs. With the continuous progress in aerospace engineering, the error probability must be smaller and smaller, so the method in this paper has a strong application prospect.

## 4. Discussion

Although the mixture simulation method is adopted in this paper, the method proposed in this paper is suitable for any hierarchy, and it can also be used to improve the speed of system-level simulation in cases where the time cost is more concerned. In this paper, 21 checkpoints are added to one workload execution to determine error checkpoints. This is because the mixture simulation takes several minutes to complete one mixture workload execution. Adding 21 checkpoints will not increase the time particularly. However, the number of checkpoints needs to be reduced if the method is applied to system-level simulation because the system-level simulation time is shorter than the mixture simulation method. Adding too many checkpoints may reduce simulation efficiency. 

The time interval between the two checkpoints proposed in this paper is determined by the time the processor completes one process from reading data to writing data, related to the test program. A more general method to select the time interval will be studied in the future. This method will be applied to multiple-bit upset (MBU) in future work.

## 5. Conclusions

Simulating fault injection using computer simulation techniques is an effective way to reflect the SEE in aerospace integrated circuits. This paper proposes a multi-point fault injection method to achieve a good acceleration effect. Further, this method does not make the accuracy decline. Simulation results based on the Leon2 system validated the correctness of the proposed approach and showed that the proposed method could achieve a speedup of 60.69× at most. 

## Figures and Tables

**Table 1:** Relevant simulation parameters.

Setup	Parameters	Description
Time interval	300 ns	Analyze the test program
Injection time	8700–15,000 ns	From the end of the system initialization
LET	100 MeV·cm^2^/mg	Make sure errors can be detected

**Table 2:** Areas of different modules.

Fault Injection Module	$\mathrm{Area}/\mu m^{2}$
Integer unit	53,148.99
Cache	21,611.30
Memory control module	12,165.27
Register-file	421,410.11

**Table 3:** Speedup of different injection modules with and without checkpoints.

Fault Injection Module	Error Probability	Speedup without Checkpoints	Speedup with Checkpoints
Integer unit	2.85%	3.88×	4.17×
Cache	0.82%	9.81×	10.30×
Memory control module	1.92%	5.16×	5.34×
Register-file	0.09%	57.56×	60.69×

**Table 4:** The optimal number and grouping method of different injection modules and corresponding speedup by theoretical analysis.

Fault Injection Module	The Optimal Number of Faults	The Optimal Grouping Method	Speedup by Theoretical Analysis
Integer unit	27	3	3.90×
Cache	81	3	9.92×
Memory control module	27	3	5.21×
Register-file	729	3	61.43×

**Table 5:** The number of faults that need to be searched under different numbers of error faults with and without addition of checkpoints after the error probability is stable.

Injection Module	Error Faults = 1		Error Faults = 2		Error Faults ≥ 3	
	With Checkpoints	Without Checkpoints	With Checkpoints	Without Checkpoints	With Checkpoints	Without Checkpoints
Integer unit	11.81	27	14.49	27	16.71	27
Cache	29.88	81	44.65	81	57.31	81
Memory control module	14.63	27	17.24	27	19.28	27
Register-file	234.25	729	348.45	729	443.33	729

**Table 6:** The probabilities of different numbers of error faults.

Injection Module	The Probability of Error Fault = 0	The Probability of Error Fault = 1	The Probability of Error Fault = 2	The Probability
				of Error Fault ≥ 3
Integer unit	45.13%	39.82%	9.73%	5.32%
Cache	51.30%	33.91%	12.17%	2.62%
Memory control module	60.55%	29.05%	8.26%	2.14%
Register-file	53.15%	28.83%	13.51%	4.51%

## Data Availability

Not applicable.
